# Causal effects of challenge and threat appraisals on pain self-efficacy, pain coping, and tolerance for laboratory pain: An experimental path analysis study

**DOI:** 10.1371/journal.pone.0215087

**Published:** 2019-04-23

**Authors:** Shuanghong Chen, Todd Jackson

**Affiliations:** 1 Key Laboratory of Cognition & Personality Southwest University, Chongqing, China; 2 Department of Psychology, University of Macau, Taipa, Macau S.A.R; Universiti Sains Malaysia, MALAYSIA

## Abstract

**Background:**

Primary appraisals of pain as a potential threat influence pain perception and coping but comparatively less is known about related effects of challenge appraisals or causal effects of primary appraisals on secondary appraisals of perceived pain coping capacities (e.g., pain self-efficacy).

**Methods:**

To address these gaps, young Chinese women (N = 147) and men (N = 88) were randomly assigned to one of three appraisal conditions prior to engaging in a cold pressor test (CPT): (1) a higher threat appraisal condition featuring task orienting information describing symptoms and consequences of frostbite, (2) a lower threat appraisal condition featuring orienting information about safety of the CPT, or (3) a challenge appraisal condition featuring orienting information describing benefits of persistence despite discomfort for future life satisfaction.

**Results:**

Compared to peers in the higher threat condition, challenge condition cohorts experienced smaller decreases in task-related self-efficacy, more cognitive coping, and less catastrophizing as well as more overall pain tolerance. A path analysis indicated that self-efficacy changes, cognitive coping and catastrophizing fully mediated links of subjective challenge and threat appraisals with pain tolerance.

**Conclusions:**

Initial appraisals about the nature of pain influence, not only pain tolerance and coping, but also perceived coping capacities.

**Perspective:**

Through examining particular theory-based pain appraisal and coping processes, this experiment is the first to identify both perceived coping capacities (pain self-efficacy) and coping responses as factors that explain why appraisals of pain as a challenge or threat have differential effects on pain tolerance.

## Introduction

Based on the transactional model [1,2], primary appraisals or initial judgments of stressors as a source of threat (i.e. potential damage) or challenge (i.e. opportunities for future profit, growth or development) influence “secondary” appraisals reflecting perceived coping capacities and coping options from which to choose as well as coping efforts made in the service of managing stressors. As applied to pain, certain transactional model premises have garnered support while others are not well understood. This experiment was designed to provide a more complete test of transactional account assumptions in a laboratory pain sample.

In a recent meta-analysis on the impact of primary appraisals on functioning within 59 samples with chronic pain (n = 9135), threat appraisals of pain as a source of potential tissue damage were associated with significant elevations in pain severity, impairment, emotional distress, and passive coping [3]. In the same paper, a meta-analysis of 22 laboratory pain studies (n = 2031) indicated exposure to threat appraisal manipulations caused significant overall decreases in pain tolerance and increases in passive coping (e.g. catastrophizing).

Comparatively few studies have examined challenge appraisals of pain. Unruh and Ritchie [4] were the first to create a pain-specific scale that conceptualized challenge appraisals as involving tests of strength, endurance or ability vis a vis pain events with the potential for growth, mastery or gain. Their work and that of subsequent chronic pain research found positive overall associations of challenge appraisals with active coping and negative overall relations with impairment, emotional distress and passive coping. Self-reported challenge appraisals also have a positive overall association with pain tolerance and a non-significant correlation with intensity of laboratory pain based on a recent meta-analysis [3].

Unfortunately, causal effects of challenge appraisals on responses to laboratory pain have received comparatively little consideration in the literature. However, studies that used experimental manipulations consistent with Lazarus’ [1] inclusion of opportunities for profit as an aspect of challenge appraisal suggest financial incentive opportunities increase willingness to tolerate painful stimulation compared to the absence of such inducements [5–7]. Conversely, even though challenge appraisals of suffering and pain as opportunities for growth and/or development are bedrocks of several world religions, little is known about their causal effects under controlled laboratory conditions. In the sole experimental pain study address this issue, Wang et al. [8] recently found exposure to pre-task challenge appraisal information about future psychological benefits of persevering at difficult tasks predicted higher pain tolerance, more cognitive coping (use of attention diversion, self-encouragement) and less catastrophizing among pain-free young adults undertaking a cold pressor test (CPT) compared to cohorts exposed to orienting information that was related to task safety or, especially, the threat of tissue damage.

Although these findings suggest challenge appraisals targeting future growth and development opportunities can influence pain tolerance and coping, replications are needed to ensure preliminary results are reliable. Furthermore, experimental pain studies based on the transactional model have never evaluated the central premise that primary pain appraisals affect secondary appraisals, particularly those reflecting perceived coping capacities. Self-efficacy, confidence in one’s capacity to successfully execute a given course of action [9,10], is a widely examined exemplar of perceived coping capacities [1] that has demonstrated utility in predicting less debilitating pain outcomes [11–13]. Notwithstanding correlational evidence of significant associations between primary appraisals of pain and pain self-efficacy beliefs [14–17], researchers have yet to demonstrate causal relations within experimental research designs.

To address these gaps, we tested effects of exposure to threat versus challenge appraisal manipulations of laboratory pain on changes in perceived coping capacities (i.e., pain self-efficacy), pain coping responses (catastrophizing, cognitive coping), and pain tolerance. Based on the preceding overview, compared to participants randomly assigned to lower and, especially, higher threat appraisal conditions, those assigned to a challenge appraisal condition would report more task-related self-efficacy following a standardized brief initial cold pressor test (CPT), more cognitive coping and less pain catastrophizing as well as displaying longer pain tolerance during an extended CPT. In addition, a path analysis (PA) was employed within the context of this experimental design to test whether hypothesized causal links between primary threat and challenge appraisals and pain tolerance were mediated by pain self-efficacy and pain coping.

## Method

### Participants

The final sample comprised young adults (147 women, 88 men) from Southwest University (SWU), Chongqing, China. Participants were 17 to 22 years of age (*M* = 19.32 years, *SD* = 1.23 years), typically in their first or second year of study (63%), and predominantly of Han majority ethnicity (86%), with others identifying from one of 12 ethnic minorities. All respondents were single though 26% were in a dating relationship. The sample had an average body mass index (BMI) of 20.87 (*SD* = 2.87, range: 15.94 to 30.44). As described in the preliminary results section, data from five other participants were excluded from analyses.

### Apparatus

A cold water bath unit (Model DX-208), 25-cm wide, 25-cm long, 20-cm deep and filled with 12.5 L of circulating water, was used to induce pain. A thermostat-controlled electric pump maintained water temperature (± 0.1 degrees) via heat exchange. Water temperature was maintained at 2 degrees Celsius for this experiment and a four minute time limit was set for the post-manipulation CPT. As noted in previous peer-reviewed studies from pain journals [8,18,19], the CPT is a widely-used, safe, effective method of inducing temporary discomfort. With the exception of persons who fulfill exclusion criteria, the procedure has no risk for lasting, long-term negative effects because the water temperature is maintained above the freezing point and exposure is both brief and limited to part of an extremity.

### Procedure

The current laboratory pain research featured a one factor (appraisal condition: challenge vs. higher threat vs. lower threat) between groups experimental design and was approved by the SWU Human Research Ethics Committee (ethics approval #: 534472715). Given that the study was restricted to university students, the human research ethics committee required informed consent from all participants but not parents or guardians of students who were under age 18. Potential volunteers were recruited via internet-based advertisements soliciting volunteers for a laboratory experiment on factors that influence cold water pain. Interested parties completed a checklist of exclusion criteria (i.e., presence of current pain, a current pain condition or history of past ongoing pain, blood circulation or cardiovascular disorder, epilepsy, anemia, hypertension, previous serious cold injury to hands, problems with blood clotting, skin condition, use of medications for these conditions). Appointments were made with those who remained interested, were available, and endorsed no exclusion criteria.

After arriving at their scheduled appointments, participants read and signed a written informed consent that reiterated the general research purpose, main research tasks (completion of self-report questionnaires, CPTs), time involved (about 30–40 minutes), statements regarding the right to withdraw from the study at any point one chose without penalty or loss of compensation, and compensation for participation (20 yuan). Participants completed the exclusion criteria checklist a second time followed by background measures (e.g. age, gender, height, weight, trait resilience). Based on other published work [8,20,21], a standardized 15s pre-manipulation “practice” CPT was undertaken to ensure all participants (1) were minimally pain tolerant, (2) had passing familiarity with task-related sensations prior to potentially prolonged exposure, and (3) were willing to bear pain related to an actual CPT. Specifically, the experimenter asked participants to immerse their non-writing hand in a container of room temperature water for about 30s to ensure their hand temperature was the same as that of other participants. Next, participants were told to keep the same hand in the cold water for 15s with the explicit statement that nearly everyone has been able to do so in past experiments. Indeed, all participants successfully completed the practice CPT in this study.

Following this task, participants completed self-report scales described below and a task-specific self-efficacy scale (SES) that assessed perceived confidence in their capacity to successfully tolerate pain from the upcoming CPT. Subsequently, they read one of three previously-validated orienting passages about the CPT [8,18,20]. Participants were randomly assigned to one of three conditions via a random number generator in Excel. Those randomly assigned to the higher threat condition read a factual orienting passage describing symptoms of frostbite (tingling, numbness, loss of feeling, reduced mobility) as a result of prolonged exposure to cold and its effects (e.g., waxy skin, skin turning blue, gangrene, amputation) to underscore pain as a warning for possible tissue damage. Those randomly assigned to the lower threat condition read a factual orienting passage describing the use and safety of the CPT within various groups (i.e., the task typically causes pain but not tissue damage). Based on recent work [8], those randomly assigned to the challenge condition read a factual orienting passage highlighting research on perseverance at difficult laboratory tasks as a predictor of success and well-being 30 years later and the CPT as an opportunity to display persistence at challenging tasks. Concluding sections of the passages were identical and stated that participants should try to leave their hand in the ice water for as long as possible but to remove it whenever they wanted, particularly if sensations were too uncomfortable. Orienting passages were matched for length (i.e., 359 Chinese characters).

After reading the orienting passage to which they had been assigned, participants completed a standardized manipulation check item in which they were to correctly identify the main point of the passage they had just read from the correct choice and “two incorrect distractors”. Those who failed to identify the correct passage for their experimental condition had to re-read their orienting passage and answer the question correctly before continuing. Next, participants completed items tapping task-related threat and challenge appraisals of the CPT [8] and were re-administered the task-specific SES. Instructions for the second CPT were the same as those from the initial CPT, except that participants were told to leave their hand in the ice water for as long as possible (to a four minute maximum of which they were not informed [8,18,19]).

Participants were told they could cope any way they chose during the CPT, though the experimenter would remain out of sight (behind them) and would not speak with them again until the task was terminated. Following the CPT, participants completed an adapted task-specific Coping Strategies Questionnaire [18,19]. They were then asked to guess the main hypotheses, were debriefed about the main research focus, and paid for participation.

### Measures

#### Demographics

Gender, age, relationship status, ethnicity, height, weight and total years of university education were assessed.

#### Connor-Davidson Resilience Scale–Chinese (CDRS-C)[22]

To evaluate and rule out group differences in the pre-task capacities to successfully adapt to adversity, participants completed the 10-item CDRS-C. Items were rated between *0* = “*never*” and *4* = “*almost always*” with higher total scores indicating more resilience. The original CDRS factor structure was replicated and is stable (*r* = .90 over two weeks) in Chinese respondents [21]. In this sample, the CDRS-C alpha was *α* = .87.

#### Task Specific Self-Efficacy Scale (SES)[23]

A four-item SES assessed perceived confidence in tolerating pain during the CPT based on a longer 10-item version [23]. Selected items assessed confidence in being able to “leave your hand at the icy water for a little while at least”, “bear mild pain for a short period of time”, “bear a medium amount of discomfort during the CPT” and “continue the CPT even with severe pain”. Following other published work [24,25], degree of confidence for each statement was assessed between *0 = not at all confident* and *100 = completely confident*. SES alphas were *α* = .74, and *α* = .75 for pre- and post-orienting passage exposure assessments.

#### Subjective Threat and Challenge Appraisal Scale (STCAS)[8]

The 6-item STCAS examined appraisals of the CPT as a potential threat (4 items) and challenge (2 items). All items were rated between *0 = strongly disagree* and *4 = strongly agree* with higher total scores reflecting more intense appraisals. A two component structure reflecting threat and challenge appraisals was found in validation research [8]. Alphas were *α* = .70 and *α* = .81 for threat and challenge appraisal subscales, respectively, in this sample.

#### Adapted Coping Strategies Questionnaire (ACSQ)[8,18,19]

Six diverting attention items, 5 coping self-statement items, 5 ignoring pain items, and 4 catastrophizing items were adapted from the original CSQ [26] to assess cognitive coping and catastrophizing reported “during the cold pressor test”. Items were rated between *1 = never did that* and *6 = very often did that*. Factor analyses of U.S.- and China-based studies have found a two-component structure for these items with one factor comprising all pain catastrophizing items and the other “cognitive coping” comprising all attention diversion, coping self-statements, and ignoring items [8,18]. In this study, ACSQ alphas were *α* = .88 for cognitive coping component and *α* = .84 for catastrophizing.

#### Pain tolerance

Pain tolerance was defined as total time immersed in the ice water, up to a 4 minute maximum.

### Data analysis

SPSS 20.0 was used for analyses of variance (ANOVAs) and bivariate correlation analyses. Initial univariate ANOVAs assessed appraisal condition differences on background measures (i.e., demographics, CDRS-C scores, pre-manipulation task-related self-efficacy). Subsequently, a multivariate analysis of variance (MANOVA) assessed differences on self-report measures of subjective threat and challenge appraisals, pre- to post-manipulation exposure changes in task-specific SES, and task-related cognitive coping and pain catastrophizing.

Within the entire sample, preliminary bivariate correlation analyses were run to identify correlates of pain tolerance. Next, Mplus7.4 was used to assess a model of causal links between subjective primary appraisals of threat and challenge, changes in secondary appraisal (pain self-efficacy), reported pain coping, and pain tolerance. Although linear causal modeling based on the three different appraisal conditions was possible using a multi-categorical indicator coding method [27], the approach has notable disadvantages in that 1) it does not permit the assessment of individual differences in *perceived* task-related challenge versus threat appraisals when using control group participants as the reference group or 2) tests of mediating effects based on results of bootstrapping iterated thousands of times regardless of current model fit indices. Consequently, continuous measures of subjective threat and challenge appraisals were used in analyses instead of parameter estimates based on inclusion within one of the three broad primary appraisal conditions.

Observed variables in the PA were represented by rectangles. Standard residual variance in each exogenous variable was represented by small circles. Unidirectional lines with one arrow represented hypothesized direct causal relations between two variables; those with “receiving” arrows were characterized as dependent variables. Conversely, bidirectional lines with arrows at each end reflected unanalyzed covariance between two variables and implied no direction of effect. Considering possible minor deviations from multivariate normality based on skewness and kurtosis, parameter estimates were calculated via the maximum likelihood method [28]. Following recommendations of Jackson et al. [17], hypothesized model fits were assessed with the Comparative Fit Index (CFI) and Tucker- Lewis Index (TLI) as incremental measures, the Root Mean Square Error of Approximation (RMSEA) and Standardized Root Mean Squared Residual (SRMR) as residuals-based fit indices, and the chi-square, associated degrees of freedom (CMIN/*df* ratio), and p-value. Acceptable fit thresholds for CFI (.95), TLI (.95), RMSEA (.05-.08), SRMR (less than .05), CMIN/*df* (2.0–5.0), and p-value (more than .05) were based on recommendations of past research [29–31].

#### Sample size

G*Power software (free download: www.gpower.hhu.de) generated a sample size estimate based on the use of one-way ANOVAs. Based on effect size confidence intervals in the small to medium range in a previous meta-analysis of pain appraisal manipulation experiments [3] and effect sizes for associations of threat (*r* = -.23) and challenge appraisals (*r* = .21) with pain appraisals in the research on which this study was based [8], a minimum sample N of 228 (76 participants per group) was needed to detect an effect size of .22 at 85% power with an error rate probability of *p* = 0.05. This estimate was consistent with Kline’s [29] contention that a sample N of 200 is typically sufficient for causal modeling studies, albeit model complexity is an additional consideration. Assuming that up to 5% of data might not be usable following data collection, we sought to recruit approximately 240 participants for the study.

## Results

### Preliminary analyses

#### Data screening and manipulation checks

Data from one man in the higher threat condition who had completed a previous CPT study were omitted from analyses. Based on manipulation checks, data from one man and one woman in the higher threat condition, one woman in the lower threat condition, and one challenge condition woman were excluded because they had correctly guessed hypotheses and their responses may have reflected demand characteristics.

#### Appraisal condition differences on background characteristics

Appraisal conditions did not differ on participant gender χ^2^ (2, 235) = .06, *p* = .972, relationship status, χ^2^ (2, 235) = .34, *p* = .844, or ethnicity χ^2^ (2, 235) = 1.18, *p* = .556. No appraisal group differences were observed for age, *F* (2, 232) = 0.42, *p* = .658, year of study, *F* (2, 232) = 1.40, *p* = .249, BMI, *F* (2,232) = 1.26, *p* = .285 or trait resilience, *F* (2, 232) = 0.31, *p* = .735. Due to the focus on task-related self-efficacy changes following exposure to primary appraisal manipulations, appraisal group differences in pre-exposure task-based self-efficacy were also assessed. There was no group differences on pre-exposure pain self-efficacy, *F* (2, 232) = 0.50, *p* = .607 [Challenge condition: *M* = 81.06 *SD* = 17.06 vs. Higher Threat condition: *M* = 81.37 *SD* = 15.11 vs. Lower Threat condition: *M* = 83.31 *SD* = 13.36].

### Main analyses

#### Appraisal condition differences in pain tolerance, primary appraisals, self-efficacy, and coping

Based on the significant multivariate effect for appraisal condition, *F* (12, 456) = 7.86, *p* < .001, univariate ANOVAs indicated challenge condition participants had significantly longer pain tolerance than peers in the higher threat condition had (*p* = .017) and marginally longer tolerance than lower threat condition controls had (*p* = .080) (see Table 1). Conversely, no tolerance differences were found between higher and lower threat condition subgroups (*p* = .505). In addition, participants in higher threat and challenge appraisal conditions, respectively, rated the task to be more subjectively threatening and challenging than did peers in complementary conditions. Higher threat condition participants also showed sharper decreases in task-related self-efficacy scores after reading their orienting passage than did peers in lower threat (*p* = .009) or challenge (*p* = .003) conditions. Conversely, the latter two groups did not differ in self-efficacy changes (*p* = .736). On ACSQ factors, challenge condition participants reported significantly more cognitive coping (*p* = .003) and less catastrophizing (*p* = .004) in managing CPT pain than did higher threat condition participants.

**Table 1:** Appraisal condition differences in subjective pain appraisals, reported pain coping and pain perception (N = 235).

	Appraisal conditions			Univariate *F*-values	
Measures	Challenge (CH) (N = 79)	Higher threat (HT) (N = 77)	Lower threat (LT) (N = 79)	*F*	*Post-hoc* comparisons
Pain tolerance [in seconds]	117.89 (86.20)	86.99 (72.42)	95.54 (80.56)	3.12*	CH > HT*^,^ CH > LT^†^
Threat appraisal	5.86 (3.14)	7.55 (3.06)	5.46 (2.63)	10.96***	HT > CH***^,^ HT > LT***
Challenge appraisal	4.73 (1.99)	2.51 (2.04)	2.48 (2.01)	32.48***	CH > HT***^,^ CH > LT***
Self-efficacy changes	-.50 (7.76)	-3.81 (7.57)	-.87 (5.31)	5.29**	CH > HT**^,^ LT > HT**
Cognitive coping	56.53 (16.21)	48.38 (17.33)	51.86 (17.68)	4.48*	CH > HT**
Catastrophizing	8.89 (5.13)	11.30 (4.90)	10.19 (5.44)	4.27*	HT > CH**

† p < 0.10

* p < 0.05

** p < 0.01

*** p < 0.001 based on two-tailed significance tests.

#### Bivariate correlates of pain tolerance

Pain tolerance had significant associations with younger age (*r* = -.23, *p* < .001) and male gender (*r* = .18, *p* = .006), but not BMI (*r* = -.06, *p* = .404) or trait psychological resilience (*r* = .09, *p* = .183). Consequently, gender and age were included as covariates in partial correlation analyses of relations between tolerance and task-specific measures of appraisal and coping (see Table 2). Pain tolerance elevations were related to lower subjective threat appraisal and catastrophizing scores as well as higher subjective challenge appraisals levels, higher task-related self-efficacy change scores, and more reported use of cognitive coping during the CPT.

**Table 2:** Partial correlations^1^ between measures of pain tolerance, appraisal and coping (N = 235).

	1.	2.	3.	4.	5.	6.
1. Pain tolerance (in seconds)	—					
2. Subjective threat appraisal	-.26***	—				
3. Subjective challenge appraisal	.15*	-.07	—			
4. Self-efficacy changes	.14*	-.13*	.21**	—		
5. Cognitive coping	.19**	-.20**	.45***	.12	—	
6. Catastrophizing	-.50***	.40***	-.06	.03	-.08	—

*p < 0.05

**p < 0.01

***p < 0.001.

Note

^1^: Controlling for gender and age.

#### Path analysis (PA) of influences on pain tolerance

**Fig 1:**
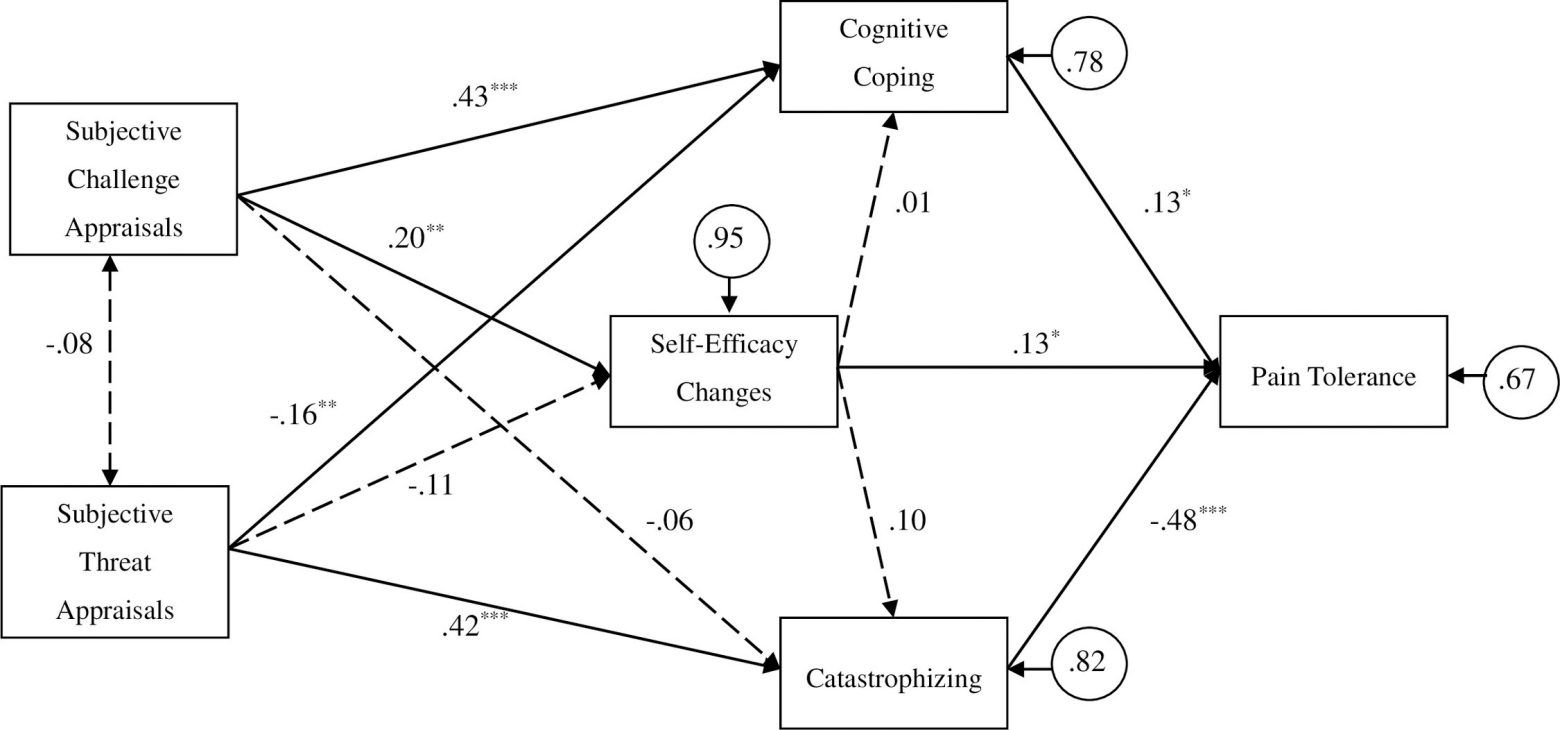
. Path analysis of associations between subjective primary appraisals of pain, self-efficacy changes, coping, and pain tolerance (N = 235). *p < 0.05; **p < 0.01; ***p < 0.001. *Note*: proportion of variance explained can be calculated via the formula: 1 –standardized error variance (symbolized by a circle). Values given in the pathways represented standardized regression coefficients. Solid and broken lines between measures, respectively, reflected significant and non-significant paths.

Hypothesized associations between primary appraisals, changes in secondary appraisal (pain self-efficacy), reported pain coping, and pain tolerance were computed within a PA. After controlling for effects of age (*β* = -.15, *p* = .005) and gender (*β* = .14, *p* = .009) on pain tolerance, the constrained PA model (Fig 1) resulted in uniformly acceptable overall model fits (CFI = .990, TLI = .977, RMSEA = .031 (90% confidence interval: 0 - .083), SRMR = .032, CMIN/*df* = 1.22, *p* = .276).

As a possible function of being randomly assigned to distinct appraisal conditions, there was a non-significant association between challenge and threat appraisals of cold pressor pain (Fig 1). However, challenge appraisal elevations were related to higher in pre- to post-manipulation self-efficacy scores and more reported use of cognitive coping, which corresponded, in turn, with increased CPT tolerance (Fig 1). Conversely, higher threat appraisal scores were related to lower pre- to post-manipulation self-efficacy scores, though the association reflected only a statistical trend (*p* = .086). Higher subjective threat appraisal levels were also related to increases and decreases in pain catastrophizing and cognitive coping, respectively. Participants who reported larger increases in pre- to post-manipulation self-efficacy, less task-related pain catastrophizing, and more cognitive coping tended to display longer pain tolerance durations (see Fig 1).

Finally, mediation effects were computed via bootstrapping iterated 1000 times. Direct paths of subjective challenge appraisals (*β* = .04, *p* = .501) and threat appraisals (*β* = -.03, *p* = .647) to pain tolerance were not significant, indicating possible mediating effects of self-efficacy changes and coping on these relations [32]. After dropping non-significant paths of subjective appraisals to pain tolerance, indirect effect sums from subjective challenge appraisal and subjective threat appraisal to pain tolerance were significant (see Table 3). Specifically, (1) self-efficacy changes mediated the association of subjective challenge appraisals to pain tolerance, (2) cognitive coping mediated the association of subjective challenge appraisals to pain tolerance, and (3) catastrophizing mediated the association of subjective threat appraisals to pain tolerance. In sum, paths of challenge and threat appraisal to pain tolerance were fully mediated by changes in self-efficacy, pain catastrophizing, and/or cognitive coping.

**Table 3:** Indirect effects of subjective pain appraisals on pain tolerance (bootstrapping iterated 1000 times).

Path	Estimate	Standard Error	*P*
Effects from challenge appraisal (CA) to pain tolerance			
Sum of indirect effects	.10	.04	.012
CA→self-efficacy changes→pain tolerance	.03	.01	.050
CA→cognitive coping→pain tolerance	.06	.03	.023
CA→self-efficacy changes→cognitive coping→pain tolerance	.00	.002	.865
CA→catastrophizing→pain tolerance	.03	.03	.401
CA→self-efficacy changes→catastrophizing→pain tolerance	-.01	.01	.198
Effects from threat appraisal (TA) to pain tolerance			
Sum of indirect effects	-.23	.04	.001
TA→self-efficacy changes→pain tolerance	-.01	.01	.224
TA→cognitive coping→pain tolerance	-.02	.01	.096
TA→self-efficacy changes→cognitive coping→pain tolerance	.00	.001	.883
TA→catastrophizing→pain tolerance	-.20	.04	.001
TA→self-efficacy changes→catastrophizing→pain tolerance	.01	.01	.384

## Discussion

Reviews of the literature have concluded exposure to threatening information about pain affects self-reported coping and behavioral pain tolerance [3]. Aside from replicating these findings, this experiment documented novel, additional effects of exposure to distinct primary appraisal manipulations. Furthermore, PA indicated that pain self-efficacy changes, cognitive coping, and pain catastrophizing fully mediated associations between primary appraisals and tolerance of cold pressor pain. Implications of main findings are elaborated below.

First, regarding effects of exposure to primary appraisal manipulations, this experiment was the first to document causal effects of primary pain appraisals on perceived pain coping capacities. Bandura [10] has pointed out that past experience with a specific task is a critical determinant of task-related self-efficacy. To control for the impact of past task-related experience in this experiment, participants were screened to ensure they had no past experience with the CPT and were then exposed to a brief standardized 15s CPT that they all completed successfully. Coupled with the use of random assignment to manipulations, these steps helped to ensure appraisal conditions were equated on experience with the CPT prior to being exposed to their respective orienting passages. Reassuringly, participants assigned to higher threat, lower threat and challenge appraisal conditions were not differentiated on the basis of pre-manipulation task-related self-efficacy levels. Consequently, any appraisal condition differences in self-efficacy that emerged following exposure to appraisal manipulations were attributable to differences in primary appraisal orienting information.

In line with general transactional model premises that primary appraisals regarding the nature of stressors influence secondary appraisals of perceived capacities to cope with stressors [2] and Bandura’s [10] contention that verbal persuasion and building awareness of physiological and affective states before and after performing desired activities are alternative means of influencing SE, exposure to higher threat orienting information resulted in significant losses of pain self-efficacy compared to viewing orienting passages that reflected either challenge appraisals (i.e., persisting in the face of discomfort as a predictor of future life satisfaction) or reduced threat (i.e., task-related pain as uncomfortable but not damaging). Recent correlational studies of chronic pain samples have also linked threat and challenge appraisals to pain self-efficacy [14, 15]. However, the experimental design used here unambiguously established causal effects of primary appraisals on changes in pain self-efficacy beliefs. Presumably, because self-efficacy has robust associations with improved functioning in the clinical pain literature [11,12], assessing and altering appraisals of pain meanings should have utility, not only for how people cope with pain, but also for bolstering confidence in the belief that they are capable of functioning well, despite ongoing pain.

By and large, effects of exposure to the higher threat manipulation aligned with past evidence of comparatively reduced cognitive coping, increased pain catastrophizing, and/or attenuated pain tolerance in higher threat contexts [8,18–20, 33]. As expected, participants randomly assigned to the complementary challenge appraisal condition reported the highest and lowest average levels of cognitive coping and pain catastrophizing, respectively, as well as the longest mean tolerance time of any appraisal condition. These differences were all significant for challenge versus higher threat appraisal condition comparisons but not challenge vs. lower threat comparisons, largely replicating results of Wang et al. [8] who used the same challenge appraisal manipulation. Relative advantages and disadvantages of exposure to challenge versus higher threat orienting passages illustrated how being exposed to particular kinds of *external* information about the meaning of pain influences specific subjective appraisals about pain meanings and has causal effects on confidence in one’s capacity to manage pain, particular coping responses, and actual pain tolerance.

Complementing causal effects of exposure to particular external orienting passages, PA results directly underscored paths from *subjective* appraisals of pain to behavioral tolerance of pain. Individual differences in subjective challenge appraisals were linked to relative (1) elevations in confidence about one’s capacity to manage pain and (2) more frequent use of cognitive pain coping strategies (i.e., coping self-statements, ignoring, diverting attention). Cross-sectional structural equation modeling (SEM) research on chronic back pain has also linked challenge appraisals to pain self-efficacy elevations but not pain catastrophizing [14] while the significant challenge appraisal—cognitive coping path replicates that of another recent PA study [8]. PA was performed within an experimental design that tested causal effects of exposure to distinct primary appraisal manipulations. As such, claims about causal effects of challenge (and threat) appraisals on self-efficacy changes, coping responses and behavioral pain tolerance were more strongly substantiated than such claims could be if SEM had been performed within a cross-sectional, non-experimental design. Mediation analyses supported changes in pain self-efficacy and elevations in cognitive coping as possible mechanisms by which viewing pain as a challenge and future opportunity contributed to improved behavioral pain tolerance.

The PA also indicated mediation of the threat appraisal-pain tolerance path was somewhat distinct. Dovetailing with results of experiments within American [18] and Australian [20] samples, participants who viewed task-related pain as especially threatening endorsed more pain catastrophizing and less cognitive coping during the CPT. In line with early evidence [20], mediation analyses suggested possible increases in pain catastrophizing in tandem with decreases in cognitive coping explain why heightened primary appraisals of pain as a threat hinder behavioral pain tolerance. Coupled with challenge appraisal results and similar effects observed in chronic pain samples [14,15,34], these findings also highlight how pain appraisals merit consideration as one focus of initial psychological assessments of pain. Because primary appraisals of pain have causal repercussions “downstream” for managing pain in laboratory and clinical contexts [3], effects of interventions to reduce excessive threat appraisals and/or bolster challenge appraisals of pain should be evaluated.

Despite its possible implications for theory and practice, three limitations of this experiment warrant discussion. First, randomly assigning non-clinical respondents to distinct appraisal manipulations established clear causal connections between primary pain appraisals, task-related changes on pain self-efficacy, pain coping and behavioral pain tolerance, independent of potential confounds such as ongoing pain and affective distress. Notwithstanding such strengths to internal validity, extensions based on clinical pain samples and other age cohorts are needed to evaluate the external validity of results further. Second, although the coping measure used in this study includes catastrophizing as a factor [26], researchers have debated the status of this construct as a measure of primary appraisal versus secondary appraisal versus coping [35,36]. Arguably, expressions of catastrophic thinking can reflect coping efforts via venting or soliciting others’ social support though we should also acknowledge that catastrophizing can also reflect secondary appraisals of one’s perceived incapacity for coping.

Third, even though we established causal links between primary appraisals and pain self-efficacy changes [1], aside from perceived coping capacities, secondary appraisal facets include consideration of and selection from coping options; these processes were not assessed, in part, because there are no validated measures of these constructs in the pain literature. The final PA model indicated perceived confidence in the capacity to tolerate task-related pain (i.e., self-efficacy) had little to do with reported use of cognitive coping or pain catastrophizing. However, in itself, this finding does not refute the transactional model premise that secondary appraisals influence coping efforts, given that perceived coping options were not assessed. The use of alternative paradigms such as “think aloud” protocols in future research is one strategy that may elucidate coping selection processes while capturing possible moment to moment shifts in the interplay of appraisals, coping responses, and reappraisals.

## Conclusions

To our knowledge, this experiment was the first to test causal effects of primary challenge and threat appraisals of pain on secondary appraisal facets of perceived coping capacities (i.e., changes in pain self-efficacy) as well as coping and behavioral tolerance of pain. As such, the research evaluated transactional model premises that had been untested previously in relation to experimental pain [1]. PA indicated paths from subjective challenge appraisals to behavioral pain tolerance were fully mediated by pain self-efficacy changes and cognitive coping. Conversely, the threat appraisal-tolerance path was fully mediated by task-related pain catastrophizing and cognitive coping. As such, beliefs about what pain signifies (threat vs. challenge) are one key influence on beliefs about one’s capacity to successfully cope with pain and strategies that one adopts in managing pain.

## Supporting information

S1 File Full data set. (SAV)
